# Different thresholds of tissue-specific dose-responses to growth hormone in short prepubertal children

**DOI:** 10.1186/1472-6823-12-26

**Published:** 2012-11-01

**Authors:** Ralph Decker, Anders Nygren, Berit Kriström, Andreas FM Nierop, Jan Gustafsson, Kerstin Albertsson-Wikland, Jovanna Dahlgren

**Affiliations:** 1 Göteborg Pediatric Growth Research Centre (GP-GRC), Department of Pediatrics, Institute of Clinical Sciences, The Sahlgrenska Academy at University of Gothenburg, Gothenburg, Sweden; 2 Institute of Clinical Sciences, Department of Pediatrics, Umeå University, Umeå, Sweden; 3 Muvara bv, Multivariate Analysis of Research Data, Leiderdorp, Netherlands; 4 Department of Women’s and Children’s Health, Uppsala University, Uppsala, Sweden

**Keywords:** GH deficiency, GH sensitivity, GH responsiveness, Idiopathic short stature, GH dose-effect, Metabolic effects, Lipolysis

## Abstract

**Background:**

In addition to stimulating linear growth in children, growth hormone (GH) influences metabolism and body composition. These effects should be considered when individualizing GH treatment as dose-dependent changes in metabolic markers have been reported. ***Hypothesis***: There are different dose-dependent thresholds for metabolic effects in response to GH treatment.

**Method:**

A randomized, prospective, multicentre trial TRN 98-0198-003 was performed for a 2-year catch-up growth period, with two treatment regimens (a) individualized GH dose including six different dose groups ranging from 17–100 μg/kg/day (n=87) and (b) fixed GH dose of 43 μg/kg/day (n=41). The individualized GH dose group was used for finding dose–response effects, where the effective GH dose (ED 50%) required to achieve 50% Δ effect was calculated with piecewise linear regressions.

**Results:**

Different thresholds for the GH dose were found for the metabolic effects. The GH dose to achieve half of a given effect (ED 50%, with 90% confidence interval) was calculated as 33(±24.4) μg/kg/day for Δ left ventricular diastolic diameter (cm), 39(±24.5) μg/kg/day for Δ alkaline phosphatase (μkat/L), 47(±43.5) μg/kg/day for Δ lean soft tissue (SDS), 48(±35.7) μg/kg/day for Δ insulin (mU/L), 51(±47.6) μg/kg/day for Δ height (SDS), and 57(±52.7) μg/kg/day for Δ insulin-like growth factor I (IGF-I) SDS. Even though lipolysis was seen in all subjects, there was no dose–response effect for Δ fat mass (SDS) or Δ leptin ng/ml in the dose range studied. None of the metabolic effects presented here were related to the dose selection procedure in the trial.

**Conclusions:**

Dose-dependent thresholds were observed for different GH effects, with cardiac tissue being the most responsive and level of IGF-I the least responsive. The level of insulin was more responsive than that of IGF-I, with the threshold effect for height in the interval between.

## Background

In addition to stimulating linear growth in children, growth hormone (GH) influences metabolism and body composition. For several decades it has been known, based on results of *in vitro* and *in vivo* studies, that GH has effects on amino acid transport, protein synthesis [1-3], lipolysis [4,5] and glucose metabolism [2,6,7] after targeting receptors at different tissues [8]. In addition, several studies have evaluated metabolic responses to GH treatment [9-16]. GH replacement therapy in children induces favourable changes in metabolic indices and improves body composition, bone density and remodelling, physical and cardiac performance, as well as overall quality of life [12-17]. If individual responsiveness is taken into account, the metabolic response to GH treatment does not differ markedly between short children with and without classic GH deficiency (GHD) [18]. The data at baseline has previously been published [19]. It has been discussed whether any cut-off level of GH secretion chosen to distinguish between children with GHD and idiopathic short stature (ISS) may be arbitrary [19,20].

Dose-dependency with regard to GH has only been studied in a few clinical trials. It has been shown that GH treatment increases adult height in a dose-dependent manner in children with ISS [21]. GH dose-dependency of metabolic variables has not been clearly defined in short children owing to a lack of controlled trials on metabolic outcomes in response to different GH doses.

In a randomized, controlled, clinical trial we have recently shown that the anabolic and lipolytic effects of GH can be dissociated in a GH dose range between 17 and 100 μg/kg/day. Anabolism was found to be dose-dependent while lipolysis was not [18], despite a lipolytic effect being noted in all subjects. It was interpreted that the lipolytic effect of GH was overridden in the dose range used, and that dose-dependency may therefore become apparent at lower doses.

The objective of the present study was to investigate and compare the GH doses required to achieve different metabolic responses. The hypothesis was that there are dose-dependent thresholds for different tissues and metabolic functions. It was expected that the results would provide insight into the effective GH dose required to influence metabolic processes and cardiac tissue in short children with different GH secretion capacities and GH responsiveness. The key question was ‘What is the appropriate GH dose in prepubertal children to compensate for deteriorations in body composition and to avoid unfavourable metabolic effects?’

## Subjects and methods

### Ethical consideration

The study protocol (TRN 98-0198-003) was approved by the Ethics Committees of the Universities of Göteborg (for Göteborg and Halmstad), Umeå, Uppsala and Malmö and the Medical Product Agency of Sweden. Written informed consent was obtained from all parents and from children if possible. The study was performed in accordance with the Declaration of Helsinki and Good Clinical Practice.

### Subjects and study protocol

The study was a 2-year prospective, randomized, open-label, multicentre trial in short prepubertal children with isolated GHD or ISS [22] naïve to GH treatment. Individual GH responsiveness was estimated by our growth response prediction model for children with GHD and ISS [23], and the patients were randomized in 1:2 proportions to receive either a standard or an individualized GH dose during 2 years of catch-up growth to a preset growth target, mid-parental height SDS (MPH_SDS_). Randomization variables included gender, weight_SDS_ at birth, height_SDS_ at age 1 year, GH_max_AITT (during an arginine–insulin tolerance test), GH_max_24h profile (during 24h spontaneous GH sampling), age and height_SDS_ at start, the child’s height_SDS_ difference to its MPH_SDS_ (diff MPH_SDS_) at start, and predicted 1^st^ year Δ height_SDS_[23]. No patients with syndromes, chronic diseases or complete GH insensitivity were included in the study population.

Upon inclusion, all patients had a height_SDS_ below −2.0 [24] and a growth velocity below −1.0 SDS. 128 children (38 girls, 90 boys) followed the protocol [22]. Both the results from the GH_max_AITT and the GH_max_24h profile were used to separate children with GHD from those with ISS. Classic GHD was defined based on a GH_max_ below 32 mU/L (using polyclonal assay, WHO IRP 80–505) corresponding to 24 mU/L (using monoclonal assay) and equivalent to the ‘old cut-off of 10 μg/L’ [18]. According to this definition, 39 children had isolated GHD and 89 had ISS. However, when using GH_max_ results exclusively from the AITT, 90 children were assigned the diagnosis of GHD and 38 the diagnosis of ISS. Additionally, all of our patients fulfilled the criteria for GHD according to a growth velocity lesser than −1.0 SDS, IGF-I below −1 SD of sex and age specific references, bone age retardation of more than 1,5 years [18,22].

To address the question concerning GH thresholds and to study the possible dose-dependency, the analysis focused on the individualized treatment group (n=87). We used data from prepubertal children included in a trial, randomized to individual GH doses in the range of 17–100 μg/kg/d according to their growth-related GH responsiveness; with dose-adjustment for the estimated difference to the preset height target (i.e. MPH_SDS_) at 2 years on GH in order to make it possible for each child to reach its MPH_SDS_ within a 2 year period. [22]. Children with a higher predicted growth response, calculated before start of treatment, received a lower individual GH dose, and those with a lower predicted growth response received a higher GH dose. The GH doses used in the individualized-dose group were 17 μg/kg/day (n=3), 33 μg/kg/day (n=27), 40 μg/kg/day (n=10), 50 μg/kg/day (n=26), 66 μg/kg/day (n=14), and 100 μg/kg/day (n=7): the mean GH dose in this group was 49 μg/kg/day [22].

The fixed GH dose group was used as a control group for estimating a metabolic effect of the dose selection procedure in the trial by regressing the metabolic variables on the intended dose of the fixed GH dose group. The intended dose is the GH dose that the patients would have been given if they had been randomized to the group treated with individualized dose. Since this group was randomised to receive the fixed GH dose, any metabolic effect of the intended dose implicates an effect of the dose selection procedure independent of the dose given. None of the six metabolic effects appeared to be related to the dose selection procedure in the trial. Δ ALP, Δ LST_SDS_, Δ insulin and Δ IGF-I_SDS_ showed no significant effect, whereas Δ LVDd and to a lesser extend Δ height_SDS_ were even somewhat negative correlated to the intended dose of the fixed GH dose group (data not shown).

### Laboratory analyses and growth evaluation

GH and insulin-like growth factor I (IGF-I) assays were performed at the Göteborg Pediatric Growth Research Center (GP-GRC) laboratory (accredited number 1899). For analyses of serum IGF-I, leptin, insulin, alkaline phosphatase (ALP) and plasma GH, see our previous publication [18]. IGF-I was converted to SDS [25].

### Growth evaluation

Height was measured [22] and converted to SDS [24], as previously described, with use of the childhood component of the total reference [26].

### Body composition

Body composition was measured by dual-energy X-ray absorptiometry (DXA), using only one DPX-L scanner (Lunar Co., Madison, WI) at each study centre. Regular harmonization between the centres was performed following GCP/GMP. DXA assessment results in a three-compartment model of the body consisting of fat mass, lean soft tissue (LST) mass and bone mineral content (BMC). All analyses were conducted using the extended analysis program for total body analysis including bone mineral density (BMD).

### Echocardiography

Echocardiography was performed by four pediatric cardiologists and one experienced sonographer. Each child was examined longitudinally throughout the study by the same cardiologist/ sonographer. Interventricular septum thickness (IVSd), left ventricular diameter (LVDd) and left ventricular posterior wall (LVPWd) thickness were measured in diastole using M-mode. Left ventricular mass (LVM) was calculated using Devereux’s anatomically corrected formula [27].

### Statistics

For all analyses, the assumptions of normality were assessed by analysis of skewness, kurtosis and frequency histograms. A p-value of < 0.05 was considered to be statistically significant.

Delta values (Δ-values) for the metabolic variables were calculated in order to quantify changes at 2 year of treatment compared with baseline. S-shaped piecewise linear regression models were fitted with GH dose as the predictor variable and the Δ-value of the metabolic variables as response variables. It consists of 3 pieces: a horizontal head and tail and a linear piece in the middle and was plotted based on the GH-dose effect on the dependent metabolic variables. Fitting this regression comes down to fitting a bounded linear model with four parameters; the usual slope and intercept of a linear regression and an upper and lower bound for the fitted model values. The GH dose effect was given by the maximum range of the fitted piecewise function, which is equal to the difference between the fitted upper and lower bound. Half of the GH dose effect on the respective variables was calculated based on the value halfway between the lowest and highest level of the piecewise function (50% Δ effect). The number of cases within each dose group was taken into account by weighting. Corresponding 90% confidence bands were calculated.

The fitted upper and lower bounds minus the fitted intercept, divided by the fitted slope provide the two breakpoints of the predictor variable separating the middle linear part from the horizontal head and tail. The ED 50% required to achieve (50% Δ effect) is located halfway the two breakpoints of the predictor variable and a corresponding 90% confidence interval is computed. We considered the ED 50% values of pairs of metabolic variables as significantly different if their ED50% values were mutually outside each other's 90% confidence interval, each providing a 5% significance level one-sided. This ED 50% is halfway the two breakpoints of the predictor variable separating the middle linear part from the horizontal head and tail.

A one-way analysis of variance (ANOVA) with GH dose as a bounded continuous predictor was performed to test the piecewise linear GH effect. A non-parametric comparison of group means (robust test of equality of means – Welch test and Brown–Forsythe test) was conducted when variances of dependent variables were not equal across groups. To examine the influence from the 17 μg/kg/day dose group consisting of only three children, analysis were repeated with these children excluded. No significant differences resulted compared to the the complete study population. Only data consistently significant were reported.

Statistical analyses were performed with SPSS 17.0 (SPSS Inc., Chicago, USA) and with Matlab version 7.13.0 (R2011b, The Mathworks, Natick, MA, USA).

## Results

### Dose-dependency

**Figure 1:**
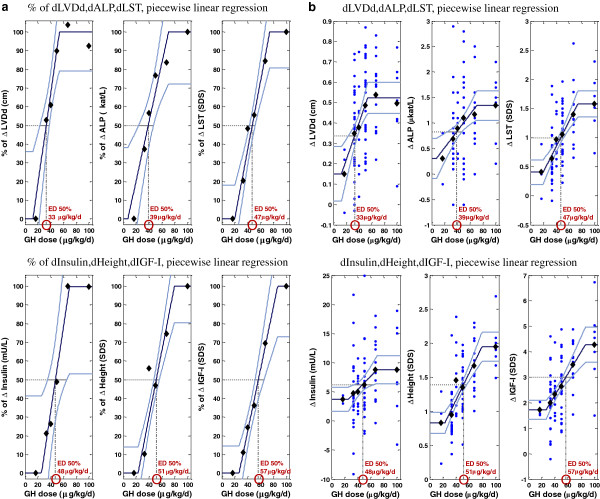
**a.Dose–response relationship of outcome variables – relative changes.** Dose–response relationships between metabolic outcome variables fitted with S-shaped piecewise linear regression lines with corresponding 90% confidence intervals. The y-axis is scaled as percent (%) of the maximum range of the fitted piecewise function. The lowest level of the piecewise function is set to 0% and the highest to 100%. The diamonds indicate the percentage change (Δ) in dose-group means (between start and 2 years) of the metabolic variables on the y-axis vs GH dose on the x-axis. LVDd: Left ventricular diameter in diastole, ALP: alkaline phosphatase, LST: lean soft tissue, IGF-I: insulin-like growth factor I. The effective GH dose (ED 50%) required to achieve half of the dose effect is calculated according to the linear regression equation of the middle part of the piecewise linear GH dose effect. **b**.Dose–response relationship of outcome variables – absolute changes. Dose–response relationship of metabolic outcome variables fitted with S-shaped piecewise linear regression lines with corresponding 90% confidence intervals. The small dots indicate the change (Δ) in response values (between start and 2 years) for 87 children receiving individualized growth hormone (GH) treatment on the y-axis vs GH dose on the x-axis. The diamonds show the dose-group means. LVDd: Left ventricular diameter in diastole, ALP: alkaline phosphatase, LST: lean soft tissue, IGF-I: insulin-like growth factor I. The effective GH dose (ED 50%) required to achieve half of the dose effect is calculated according to the linear regression equation of the middle part of the piecewise linear GH dose effect.

The variables were analyzed as Δ values between the start and 2 years of GH treatment. For each of the six dose groups, the mean Δ was calculated (Δ dose-group mean), and is shown for the 87 children receiving individualized GH treatment, Additional file 1. The dose–response relationships are shown in Figure 1. When performing ANOVA and linear regression, substantial lipolytic effects were seen in all groups as demonstrated by changes in the variables fat mass and leptin from baseline to 2 yrs of treatment; however, no dose–response differences were observed between the GH dose groups for Δ fat mass_SDS_, Δ leptin, Δ bone age, Δ BMD, Δ IVSd or Δ LVPWd (data not shown). The Δ LVM was found to be significant at the initial analysis (ANOVA p-value = 0.013) and ED 50% of 36 μg/kg/day. Excluding the small 17 μg/kg/day group, significance was lost and the data is not included in Additional file 1.

### Effective GH dose at ED 50%

Dose-dependent increases of the dose-group means of Δ LVDd, Δ ALP, Δ LST_SDS_, Δ insulin, Δ height_SDS_ and Δ IGF-I_SDS_ at 2 years of individualized GH treatment are plotted in Figure 1. All values in Figure 1a are given as percentages of the maximum range, while Figure 1b shows the same data, but now with the original scale. The lower the ED 50%, the higher is the responsiveness of a given variable. The ED 50% was lowest for Δ LVDd and highest for Δ IGF-I_SDS_ (). The ED 50% for Δ ALP, Δ LST, Δ insulin, and Δ height_SDS_ lay in the interval between these two extremes in ascending order. Differences between the Δ dose-group means for the metabolic variables were tested for a piecewise linear GH dose effect by one-way ANOVA with GH dose as a bounded continuous predictor, Additional file 1.

### Dose-responses to GH treatment

**Figure 2:**
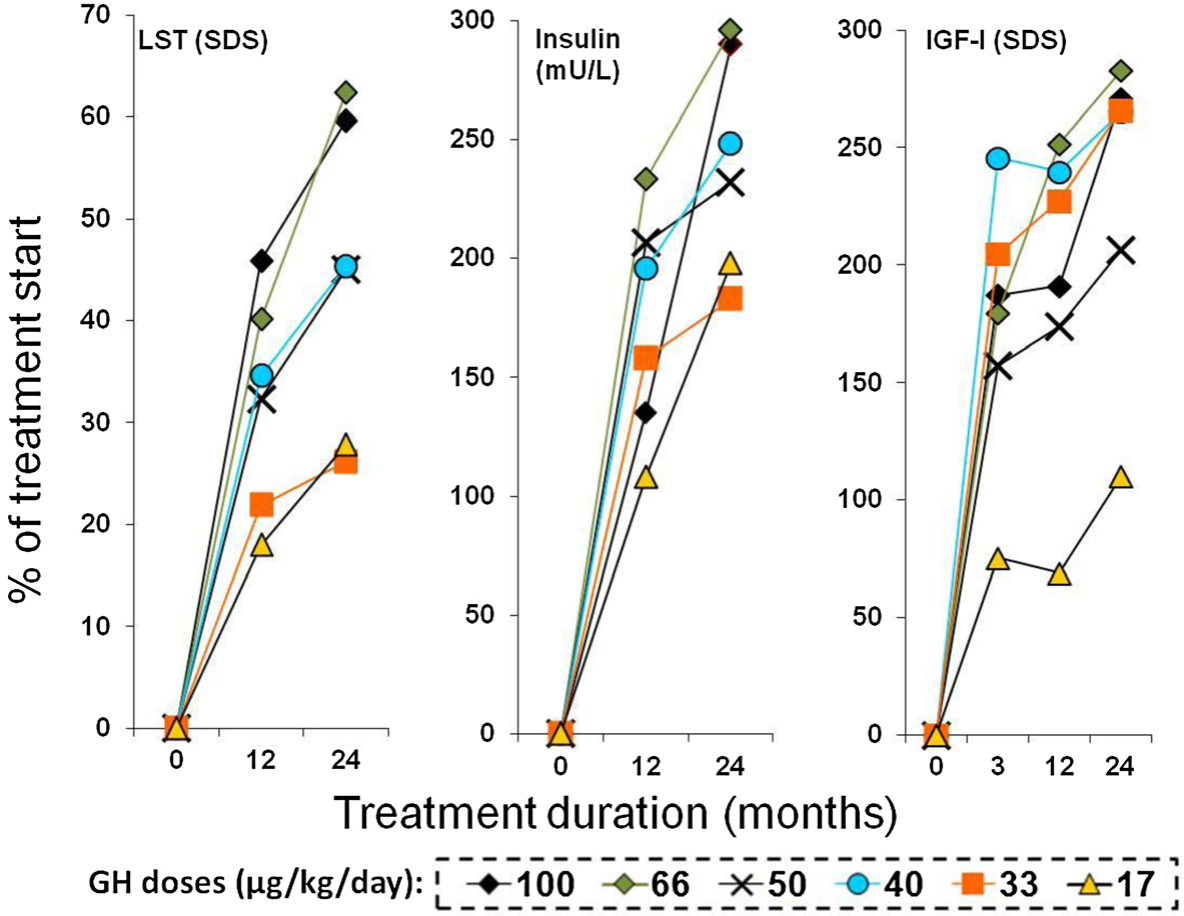
**Examples of Dose–response relationship.** Dose–response relationship for the six different growth hormone (GH) doses in 87 children receiving individualized GH treatment. The dose-group means at start of treatment are set to 0% increase from baseline values, and compared with dose group mean values at 3 months, and after 1 and 2 years of treatment. LST: Lean soft tissue, IGF-I: Insulin-like growth factor I, SDS: standard deviation score. (The absolute Δ values are presented in ref [18]).

Classic dose–response curves are depicted for the six different GH doses in the 87 children receiving individualized GH treatment, Figure 2.

Absolute dose-group means of LST_SDS_, insulin (mU/L), and IGF-I_SDS_ at start of treatment were set to 0% and compared with values from 1 and 2 years of treatment. IGF-I_SDS_ at start of treatment was also compared with values at 3 months of treatment.

Low GH doses were associated with the smallest responses. High GH doses resulted in marked responses, with an increase in LST_SDS_ by more than 60% relative to baseline and an increase of more than 250% for levels of insulin and IGF-I_SDS_ during the 2-year treatment period, Figure 2.

### Thresholds of GH dose effects

**Figure 3:**
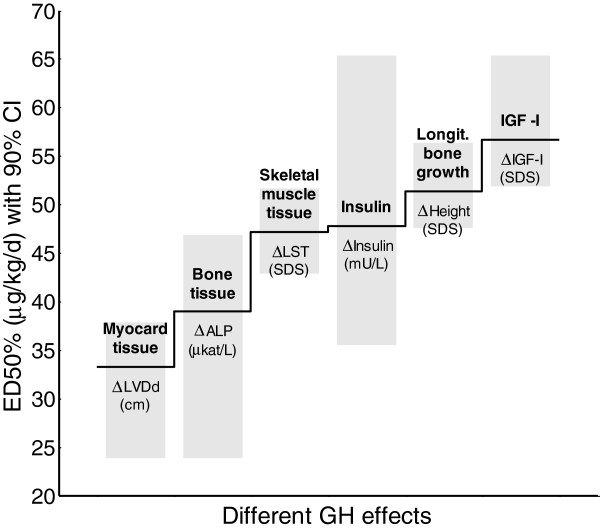
**The threshold staircase hypothesis.** The “threshold hypothesis” is presented in accordance with results from the present study as a staircase of growth hormone (GH) dose needed for an effect (no scaling). Dose–response thresholds for metabolic markers represent different metabolically active tissues or metabolic functions compared with the longitudinal growth response. LVDd: Left ventricular diameter in diastole, ALP: alkaline phosphatase, LST: lean soft tissue, IGF-I: insulin-like growth factor I. Variables are given as change (Δ) between start and at 2 years of GH treatment. The light-grey boxes mark the 90% confidence interval (CI).

An overview of the principal relationships between GH dose effects is shown in Figure 3. GH dose thresholds, correspond to the ED 50%,. are depicted as a staircase with increasing GH doses needed to achieve a certain metabolic effect. Variables are given as Δ between start and at 2 years of GH treatment.

## Discussion

In the present study, different thresholds for tissue and metabolic responses to GH treatment were found in short children who had varying GH secretion capacities, as well as varying responsiveness to GH.

### Cardiac response to GH

Cardiac tissue, estimated by LVDd, was found to be the most GH-sensitive of the variables evaluated (effects seen from a dose of 33 μg/kg/d). This is in line with the findings of Capalbo *et al*. [28] who found that LVDd increased during treatment with a dose of 30 μg/kg/d. From the available data, it is difficult to draw conclusions concerning the mechanism responsible for GH effects on heart size. We have, however, previously shown the presence of GH receptors in cardiac tissue in children, indicating that a direct effect of GH on the heart is likely [29]. Even though GH responsiveness was high, there were no adverse effects seen on any measurement of cardiac function or on blood pressure during the study [30].

### GH dose-effect on body composition

It has been shown that GH treatment at a dose of 57 μg/kg/d given to children born small for gestational age (SGA) can lead to an increase in muscle mass, and a concomitant decrease in fat mass [31]. However, data showing a dose-dependency increase in LST are lacking. We found that LST mass increased in a dose-dependent manner for the six GH doses administered. This effect was most marked at GH doses above 33 μg/kg/d, and the ED 50% for gain in LST mass was in the mid-range (47 μg/kg/d). This dose is in the same range as that found to promote longitudinal bone growth (51 μg/kg/d), confirming our previous findings of anabolic GH effects.

As demonstrated previously using principal component analysis [18], a strong lipolytic effect was found for all GH doses, seen by changes in fat mass and fat mass index, but no GH dose–response effect was seen. A possible explanation is that in the dose range studied the lipolytic effect had already reached its maximum, making lipolytic variables the most sensitive to GH. Early leptin reduction after the start of GH treatment was found previously to be positively correlated with first year growth response in a group of short children treated with 33 μg/kg/day; however, there was a wide range in Δ leptin levels as for growth response; at that time no individual responsiveness was possible to estimate [11]. It is well known that fat mass decreases in GH-deficient adults when they are treated with GH [32]. Further studies are needed to determine the dose response for the lipolytic effects at lower doses in children.

### GH effect on alkaline phosphatase

Increased serum bone-specific ALP is known to be a reliable and early sign of increased bone metabolism and correlates to first year growth response in GH-deficient children [33]. In the present study, ALP activity was found to be more responsive than longitudinal bone growth to a given GH dose.

### GH dose-effect on insulin and insulin-sensitivity

GH doses above the common dose range of 25–35 μg/kg/d used for GH-deficient children [34], resulted in greater insulin increases than lower doses, although insulin levels did not exceed the normal range. This confirms previous studies that insulin levels are lower than normal at baseline in GH-deficient individuals [35]. In short children born SGA, no impaired GH dose–response effect on insulin has been reported in a dose range between 33 and 66 μg/kg/d [36].

GH exerts both insulin-like and insulin-antagonistic effects *in vitro*[4]. An insulin-like effect has been reported in some *in vivo* studies [37], but not in all [38]. In the present study the ED 50% for the insulin enhancement was 48 μg/kg/d, which is very close to the ED 50% of 51 μg/kg/d for height gain found in the present study. Thus, the insulin antagonist effect of GH seems to be equally or more responsive to GH than the effect on IGF-I. An explanation for this may be that we used SDS for IGF-I, but not for fasting insulin, where mU/L was used. Insulin may be required as a growth factor during the catch-up growth phase, so the observation of a dose-dependent increase should be viewed as more than just compensation for GH-induced insulin resistance.

### GH dose-effect on IGF

We demonstrated a marked dose–response effect on IGF-I_SDS_. Thus, as for LST mass, the prediction of growth response is not valid for IGF-I_SDS_ levels. This is in line with findings in groups where individual responsiveness was not addressed, and GH therapy resulted in increasing IGF-I_SDS_ in a dose-dependent manner in prepubertal children, with more dramatic changes being observed at higher doses (50 and 100 μg/kg/day vs 25 μg/kg/d) [39], and 100 μg/kg/d compared to 43 μg/kg/d in pubertal GH-deficient patients [40].

In the present study, the ED 50% for IGF-I_SDS_ was 57 μg/kg/d, which was the highest dose observed for any metabolic variable. Therefore, liver response to IGF-I secretion was found to have a higher threshold than both height gain and muscle growth in the present study.

### GH dose-effect on catch-up growth

In order to be able to compare metabolism and growth, height gain was studied in the present study and not the height target of the study, i.e. the diff MPH_SDS_. The growth effect associated with individualized GH doses was quantified and presented in Figure 1. Thus, a GH dose of 51 (47, 56) μg/kg/d was necessary in order to achieve half of the height gain. In previously performed studies in children with ISS, GH had a dose-related effect on longitudinal bone growth in the dose ranges of 33–67 μg/kg/d [21] and 31–47 μg/kg/d [41]. In GH-deficient children, an increase in GH dose from 25 to 50 μg/kg/d resulted in a sustained increase in growth velocity, whereas in this study no additional effect was observed with a further increase in dose to 100 μg/kg/d, the highest GH dose in the trial given only to the most non-responsive children [39]. However, in a randomized GH dose study during puberty in GH-deficient children, a dose dependent effect (33 μg/kg/d vs. 67 μg/kg/d) was found [42]. This is in line with prior evidence for a dose-dependent effect of higher doses on adult height in children with ISS [43]. To summarize, the adult height achieved in GH-deficient children treated with GH replacement therapy has been found to be dose-dependent [34,42], as has adult height in children with ISS [21,41,43].

### Effects on metabolism

There are, however, only a few studies on the metabolic consequences of GH therapy in children. Ciresi *et al.*[12] studied metabolic parameters in GH-deficient children, but the question of dose-dependency was not investigated. Mauras *et al*. [40] compared IGF-I levels in two different GH dose groups in adolescents with GHD. Cohen *et al*. [39] analyzed the response of IGF-I, IGFBP-3, fasting glucose, fasting insulin, HbA1c and height to three different GH doses (25, 50, and 100 μg/kg/day) in prepubertal GH-deficient children. However, the question of whether there were different metabolic thresholds was neither addressed nor individual responsiveness.

In a recent report on our study group, we found dose-dependent effects on height gain, body composition and metabolism [18]. There are no comparable prospective randomized studies. Nevertheless, our data can be compared with reported qualitative effects in response to different GH doses, which suggest that dose–response effects exist [21,39].

Our study included children classified as both GHD and ISS as our study also includes aspects on tissue responsiveness, ranging from high to low within both diagnostic groups. Without a wide range, it would not have been possible to perform our study on the hypothesis of varying thresholds for different tissues and metabolic markers [19,20].

A fact that influences the interpretation of the results is, that the individual GH dose in the trial was selected based on GH responsiveness according to estimated/expected growth response and adapted so that the child would reach MPH_SDS_ within 2 years, although limited by the set maximal GH dose of 100 μg/kg/d. None of the metabolic effects presented here was found to be related to the dose selection procedure in the trial.

The strengths of the present study are that we were able to compare many different effects within the same individual, as well as assessing inter-individual variations. When monitoring GH treatment in the clinical setting, it is essential to know which processes will be affected and which marker will be the first to react to treatment based on responsiveness. The aim with estimating individual responsiveness is to set a target for treatment effect – it may be growth response for which we now have prediction models. In the future, prediction models may also be constructed for the metabolic markers studied in this paper.

## Conclusions

GH dose-dependent thresholds for different metabolic effect were found in the current study, Figure 3. Cardiac tissue was found to be the most responsive to GH treatment, followed by muscle tissue and height gain. Insulin levels incresed, reflecting GH-induced resistance and insulin was found to be more sensitive to GH than IGF-I, suggesting that insulin is as a growth factor during the catch-up growth phase in prepubertal short children.

## Abbreviations

ALP: Alkaline phosphatase; ANOVA: Analysis of variance; Apo A-II: Apolipoprotein A-II; BMC: Bone mineral content; BMD: Bone mineral density; Δ: Delta; ED 50%: Effective dose 50%; DBP: Diastolic blood pressure; DXA: Dual-energy X-ray absorptiometry; DPX-L: Proper name of the Lunar pencil beam scanner; GH: Growth hormone; GHD: Growth hormone deficiency; GP-GRC: Göteborg Pediatric Growth Research Center; HbA1c: Glycosylated haemoglobin; HDL: High-density lipoprotein; HOMA: Homeostasis model assessment of insulin resistance calculated as ((fasting serum insulin* fasting plasma glucose)/22.5); IGF-I: Insulin-like growth factor I; ISS: Idiopathic short stature; IVSd: Interventricular septal thickness in diastole; LDL: Low-density lipoprotein; Lp(a): Lipoprotein (a); LST: Lean soft tissue; LVDd: Left ventricular diameter in diastole; LVM: Left ventricular mass; LVPWd: Left ventricular posterior wall in diastole; SBP: Systolic blood pressure; SDS: Standard deviation score.

## Competing interests

R Decker and A Nygren declare that they have no competing interests. B Kriström declares that she received lecture and consultation fees. J Gustafsson declares that he owns stocks and has received consultation fee, and an unrestricted research grant from Pfizer. AFM Nierop works for Muvara bv, Multivariate Analysis of Research Data, in the Netherlands. K Albertsson-Wikland declares that she received an unrestricted research grant from Pharmacia/Pfizer until 2005 for previous studies and the present study and has received lecture fees. J Dahlgren declares that she received lecture and consultation fees, and received an unrestricted research grant from Pfizer.

## Authors’ contributions

All authors contributed intellectually to analyses and interpretation of the data; to the writing and revising of the manuscript; and have all given approval of the final version to be published. Moreover, RD took the lead of the writing and the preliminary analyses; AN contributed with the cardiac variables and further analyses; BK, as study coordinator and investigator both in Gothenburg and in Umeå; AFMN contributed with the final statistical analyses; JG as the Uppsala investigator; KAW as the initiator of the study and principle investigator and JD as a Gothenburg investigator and planned the statistical analyses.

## Pre-publication history

The pre-publication history for this paper can be accessed here:

http://www.biomedcentral.com/1472-6823/12/26/prepub

## Supplementary Material

Additional file 1 **Table S1.** Increase in studied variables in the different dose groups. Click here for file

## References

[B1] Kostyo JL Rapid effects of growth hormone on amino acid transport and protein synthesis Ann N Y Acad Sci 1968 148 2 389 407 10.1111/j.1749-6632.1968.tb20365.x 5239681

[B2] Albertsson-Wikland K Isaksson O Time course of the effect of growth hormone in vitro on amino acid and monosaccharide transport and on protein synthesis in diaphragm of young normal rats Endocrinology 1978 102 5 1445 1451 10.1210/endo-102-5-1445 744032

[B3] Hayes V Schaeffer D Mauras N Punati J Darmaun D Can glutamine and growth hormone promote protein anabolism in children with cystic fibrosis? Horm Res 2002 58 Suppl 1 21 23 12373009 10.1159/000064761

[B4] Bjorgell P Rosberg S Isaksson O Belfrage P The antilipolytic, insulin-like effect of growth hormone is caused by a net decrease of hormone-sensitive lipase phosphorylation Endocrinology 1984 115 3 1151 1156 10.1210/endo-115-3-1151 6378603

[B5] Kamel A Norgren S Elimam A Danielsson P Marcus C Effects of growth hormone treatment in obese prepubertal boys J Clin Endocrinol Metab 2000 85 4 1412 1419 10.1210/jc.85.4.1412 10770175

[B6] Goodman HM Effects of growth hormone on glucose utilization in diaphragm muscle in the absence of increased lipolysis Endocrinology 1967 81 5 1099 1103 10.1210/endo-81-5-1099 6052939

[B7] Kamel A Norgren S Lindgren AC Luthman H Arner P Marcus C Effect of growth hormone treatment on insulin action in adipocytes from children with Prader-Willi syndrome Eur J Endocrinol 1998 138 5 510 516 10.1530/eje.0.1380510 9625361

[B8] Brooks AJ Waters MJ The growth hormone receptor: mechanism of activation and clinical implications Nat Rev Endocrinol 2010 6 9 515 525 10.1038/nrendo.2010.123 20664532

[B9] Hannon TS Danadian K Suprasongsin C Arslanian SA Growth hormone treatment in adolescent males with idiopathic short stature: changes in body composition, protein, fat, and glucose metabolism J Clin Endocrinol Metab 2007 92 8 3033 3039 10.1210/jc.2007-0308 17519313

[B10] Albertsson-Wikland K Hall K Growth hormone treatment in short children: relationship between growth and serum insulin-like growth factor I and II levels J Clin Endocrinol Metab 1987 65 4 671 678 10.1210/jcem-65-4-671 3654912

[B11] Kristrom B Carlsson B Rosberg S Carlsson LM Albertsson-Wikland K Short-term changes in serum leptin levels provide a strong metabolic marker for the growth response to growth hormone treatment in children. Swedish Study Group for Growth Hormone Treatment J Clin Endocrinol Metab 1998 83 8 2735 2741 10.1210/jc.83.8.2735 9709940

[B12] Ciresi A Amato MC Criscimanna A Mattina A Vetro C Galluzzo A D'Acquisto G Giordano C Metabolic parameters and adipokine profile during GH replacement therapy in children with GH deficiency Eur J Endocrinol 2007 156 3 353 360 10.1530/eje.1.02343 17322495

[B13] Aman J Rosberg S Albertsson-Wikland K Effect of growth hormone treatment on insulin secretion and glucose metabolism in prepubertal boys with short stature Eur J Endocrinol 1994 131 3 246 250 10.1530/eje.0.1310246 7921208

[B14] Hokken-Koelega AC van Pareren Y Sas T Arends N Final height data, body composition and glucose metabolism in growth hormone-treated short children born small for gestational age Horm Res 2003 60 Suppl 3 113 114 14671407 10.1159/000074511

[B15] Roemmich JN Huerta MG Sundaresan SM Rogol AD Alterations in body composition and fat distribution in growth hormone-deficient prepubertal children during growth hormone therapy Metabolism 2001 50 5 537 547 10.1053/meta.2001.22510 11319714

[B16] Chaplin JE Kristrom B Jonsson B Hagglof B Tuvemo T Aronson AS Dahlgren J Albertsson-Wikland K Improvements in behaviour and self-esteem following growth hormone treatment in short prepubertal children Horm Res Paediatr 2011 75 4 291 303 10.1159/000322937 21304250

[B17] Carter-Su C Schwartz J Smit LS Molecular mechanism of growth hormone action Annu Rev Physiol 1996 58 187 207 10.1146/annurev.ph.58.030196.001155 8815791

[B18] Decker R Albertsson-Wikland K Kristrom B Nierop AF Gustafsson J Bosaeus I Fors H Hochberg Z Dahlgren J Metabolic outcome of GH treatment in prepubertal short children with and without classical GH deficiency Clin Endocrinol (Oxf) 2010 73 3 346 354 10.1111/j.1365-2265.2010.03812.x 20455890

[B19] Wit JM Clayton PE Rogol AD Savage MO Saenger PH Cohen P Idiopathic short stature: definition, epidemiology, and diagnostic evaluation Growth Horm IGF Res 2008 18 2 89 110 10.1016/j.ghir.2007.11.004 18182313

[B20] Albertsson-Wikland K Rosberg S Ranke M Methods of Evaluating Spontaneous Growth Hormone Secretion Functional Endocrinologic Diagnostics in Children and Adolescents 1992 76 101

[B21] Albertsson-Wikland K Aronson AS Gustafsson J Hagenas L Ivarsson SA Jonsson B Kristrom B Marcus C Nilsson KO Ritzen EM Dose-dependent effect of growth hormone on final height in children with short stature without growth hormone deficiency J Clin Endocrinol Metab 2008 93 11 4342 4350 10.1210/jc.2008-0707 18728172

[B22] Kristrom B Aronson AS Dahlgren J Gustafsson J Halldin M Ivarsson SA Nilsson NO Svensson J Tuvemo T Albertsson-Wikland K Growth hormone (GH) dosing during catch-up growth guided by individual responsiveness decreases growth response variability in prepubertal children with GH deficiency or idiopathic short stature J Clin Endocrinol Metab 2009 94 2 483 490 10.1210/jc.2008-1503 19001519

[B23] Albertsson-Wikland K Kristrom B Rosberg S Svensson B Nierop AF Validated multivariate models predicting the growth response to GH treatment in individual short children with a broad range in GH secretion capacities Pediatr Res 2000 48 4 475 484 10.1203/00006450-200010000-00010 11004238

[B24] Albertsson-Wikland K Luo ZC Niklasson A Karlberg J Swedish population-based longitudinal reference values from birth to 18 years of age for height, weight and head circumference Acta Paediatr 2002 91 7 739 754 10.1111/j.1651-2227.2002.tb03322.x 12200898

[B25] Lofqvist C Andersson E Gelander L Rosberg S Blum WF Albertsson Wikland K Reference values for IGF-I throughout childhood and adolescence: a model that accounts simultaneously for the effect of gender, age, and puberty J Clin Endocrinol Metab 2001 86 12 5870 5876 10.1210/jc.86.12.5870 11739455

[B26] Karlberg J On the construction of the infancy-childhood-puberty growth standard Acta Paediatr Scand Suppl 1989 356 26 37 2488676 10.1111/j.1651-2227.1989.tb11237.x

[B27] Devereux RB Alonso DR Lutas EM Gottlieb GJ Campo E Sachs I Reichek N Echocardiographic assessment of left ventricular hypertrophy: comparison to necropsy findings Am J Cardiol 1986 57 6 450 458 10.1016/0002-9149(86)90771-X 2936235

[B28] Capalbo D Lo Vecchio A Farina V Spinelli L Palladino A Tiano C Lettiero T Lombardi G Colao A Salerno M Subtle alterations of cardiac performance in children with growth hormone deficiency: results of a two-year prospective, case-control study J Clin Endocrinol Metab 2009 94 9 3347 3355 10.1210/jc.2008-2639 19584193

[B29] Nygren A Sunnegardh J Albertsson-Wikland K Berggren H Isgaard J Relative cardiac expression of growth hormone receptor and insulin-like growth factor-I mRNA in congenital heart disease J Endocrinol Invest 2008 31 3 196 200 18401200 10.1007/BF03345590

[B30] Nygren A Sunnegardh J Teien D Jonzon A Bjorkhem G Lindell S Albertsson-Wikland K Kristrom B Rapid cardiovascular effects of growth hormone treatment in short prepubertal children. Impact of treatment duration Clin Endocrinol (Oxf) 2012 in press. 10.1111/j.1365-2265.2012.04456.x 22651572

[B31] Schweizer R Martin DD Schonau E Ranke MB Muscle function improves during growth hormone therapy in short children born small for gestational age: results of a peripheral quantitative computed tomography study on body composition J Clin Endocrinol Metab 2008 93 8 2978 2983 10.1210/jc.2007-2600 18505766

[B32] Raben MS Hollenberg CH Effect of growth hormone on plasma fatty acids J Clin Invest 1959 38 3 484 488 10.1172/JCI103824 13641397 PMC293181

[B33] Korpal-Szczyrska M Balcerska A The effect of growth hormone treatment on serum bone alkaline phosphatase in growth hormone deficient children Pediatr Endocrinol Diabetes Metab 2008 14 4 211 214 19239787

[B34] Coelho R Brook CG Preece MA Stanhope RG Dattani MT Hindmarsh PC A randomised study of two doses of biosynthetic human growth hormone on final height of pubertal children with growth hormone deficiency Horm Res 2008 70 2 85 88 10.1159/000139149 18547954

[B35] Lippe BM Kaplan SA Golden MP Hendricks SA Scott ML Carbohydrate tolerance and insulin receptor binding in children with hypopituitarism: response after acute and chronic human growth hormone administration J Clin Endocrinol Metab 1981 53 3 507 513 10.1210/jcem-53-3-507 7021579

[B36] Van Pareren Y Mulder P Houdijk M Jansen M Reeser M Hokken-Koelega A Adult height after long-term, continuous growth hormone (GH) treatment in short children born small for gestational age: results of a randomized, double-blind, dose-response GH trial J Clin Endocrinol Metab 2003 88 8 3584 3590 10.1210/jc.2002-021172 12915640

[B37] MacGorman LR Rizza RA Gerich JE Physiological concentrations of growth hormone exert insulin-like and insulin antagonistic effects on both hepatic and extrahepatic tissues in man J Clin Endocrinol Metab 1981 53 3 556 559 10.1210/jcem-53-3-556 6114964

[B38] Moller N Jorgensen JO Schmitz O Moller J Christiansen J Alberti KG Orskov H Effects of a growth hormone pulse on total and forearm substrate fluxes in humans Am J Physiol 1990 258 1 Pt 1 E86 E91 2405702 10.1152/ajpendo.1990.258.1.E86

[B39] Cohen P Bright GM Rogol AD Kappelgaard AM Rosenfeld RG Effects of dose and gender on the growth and growth factor response to GH in GH-deficient children: implications for efficacy and safety J Clin Endocrinol Metab 2002 87 1 90 98 10.1210/jc.87.1.90 11788629

[B40] Mauras N Attie KM Reiter EO Saenger P Baptista J High dose recombinant human growth hormone (GH) treatment of GH-deficient patients in puberty increases near-final height: a randomized, multicenter trial. Genentech, Inc., Cooperative Study Group J Clin Endocrinol Metab 2000 85 10 3653 3660 10.1210/jc.85.10.3653 11061518

[B41] Wit JM Rekers-Mombarg LT Final height gain by GH therapy in children with idiopathic short stature is dose dependent J Clin Endocrinol Metab 2002 87 2 604 611 10.1210/jc.87.2.604 11836292

[B42] Albertsson-Wikland K Alm F Aronsson S Gustafsson J Hagenas L Hager A Ivarsson S Kristrom B Marcus C Moell C Effect of growth hormone (GH) during puberty in GH-deficient children: preliminary results from an ongoing randomized trial with different dose regimens Acta Paediatr Suppl 1999 88 428 80 84 10.1111/j.1651-2227.1999.tb14358.x 10102059

[B43] Wit JM Rekers-Mombarg LT Cutler GB Crowe B Beck TJ Roberts K Gill A Chaussain JL Frisch H Yturriaga R Growth hormone (GH) treatment to final height in children with idiopathic short stature: evidence for a dose effect J Pediatr 2005 146 1 45 53 10.1016/j.jpeds.2004.08.055 15644821

