# Supplementary material for: Different thresholds of tissue-specific dose-responses to growth hormone in short prepubertal children
Source: BMC Endocr Disord. 2012 Nov 1;12:26. doi: 10.1186/1472-6823-12-26 (PMC3583138; doi:10.1186/1472-6823-12-26)
Supplement: Additional file 1 — Table S1. Increase in studied variables in the different dose groups. [file 1472-6823-12-26-S1.doc]

**Table 1.**

**Figure 5**

| **Table 1 GH dose** (µg/kg/day) | Δ **LVDd** (cm) | Δ **ALP** (µkat/L) | Δ **LST** (SDS) | Δ **Insulin** (mU/L) | Δ **Height** (SDS) | Δ **IGF-I** (SDS) |
| --- | --- | --- | --- | --- | --- | --- |
| **n = 17** | 0.15 (0.10) | 0.30 (0.29) | 0.4 (0.3) | 3.7 (0.7) | 0.8 (0.3) | 1.7 (0.2) |
| **n = 33** | 0.35 (0.04) | 0.69 (0.13) | 0.6 (0.1) | 4.8 (1.2) | 0.9 (0.1) | 2.0 (0.2) |
| **n = 40** | 0.38 (0.07) | 0.89 (0.14) | 1.0 (0.1) | 5.0 (1.0) | 1.5 (0.2) | 2.3 (0.3) |
| **n = 50** | 0.49 (0.04) | 1.10 (0.17) | 1.1 (0.1) | 6.2 (1.1) | 1.4 (0.1) | 2.6 (0.2) |
| **n = 66** | 0.54 (0.05) | 1.17 (0.18) | 1.4 (0.1) | 8.8 (1.4) | 1.7 (0.1) | 3.5 (0.2) |
| **n = 100** | 0.50 (0.08) | 1.34 (0.27) | 1.6 (0.2) | 8.8 (3.1) | 2.0 (0.1) | 4.3 (0.6) |
| **50%** Δ**Effect** | **0.34** | **0.82** | **0.99** | **6.2** | **1.4** | **3** |
| **conf.bounds(90%)** | **(0.28,0.39)** | **(0.68,0.97)** | **(0.9,1.1)** | **(5.1,7.4)** | **(1.3,1.5)** | **(2.7,3.3)** |
| ***ED 50%*** | ***33*** | ***39*** | ***47*** | ***48*** | ***51*** | ***57*** |
| ***conf.bounds(90%)*** | ***(24,38)*** | ***(24,47)*** | ***(43,52)*** | ***(35,65)*** | ***(47,56)*** | ***(52,65)*** |
| *ANOVA (p-value)* | *0.00012* | *0.0024* | *<0.0001* | *0.012* | *<0.0001* | *<0.0001* |
